# Natural Snow Accumulation Gradients Mediate Environmental and Plant Trait Responses to Earlier Snowmelt in Alpine Tundra

**DOI:** 10.1111/gcb.71068

**Published:** 2026-08-24

**Authors:** Jonathan J. Henn, Katherine Bardsley, Clifton P. Bueno de Mesquita, Sarah C. Elmendorf, Nancy C. Emery, Emily C. Farrer, Chiara Forrester, Jane G. Smith, Marko J. Spasojevic, Jennifer F. Morse, Katharine N. Suding

**Affiliations:** ^1^ Department of Natural Resources and Environmental Sciences University of Illinois Urbana‐Champaign Urbana Illinois USA; ^2^ Institute for Arctic and Alpine Research University of Colorado Boulder Boulder Colorado USA; ^3^ Department of Ecology and Evolutionary Biology University of Colorado Boulder Boulder Colorado USA; ^4^ Cooperative Institute for Research in Environmental Sciences University of Colorado Boulder Boulder Colorado USA; ^5^ Department of Ecology and Evolutionary Biology Tulane University New Orleans Louisiana USA; ^6^ The Watershed Center Niwot Colorado USA; ^7^ Department of Ecology, Evolution, and Organismal Biology University of California Riverside Riverside California USA

**Keywords:** alpine plants, biotic homogenization, climate niche, early snowmelt, growing season length, microclimate, snow depth

## Abstract

Mountain ecosystems are experiencing rapid climate changes, with implications for biodiversity and ecosystem function. Changing snow dynamics, especially earlier melt, are influencing the timing and length of growing seasons, species phenology, and plant abundance. Because mountains are characterized by high topographical and microclimatic variation, effects of changing snow dynamics may depend on small‐scale variation in topographically mediated snow accumulation. Wind‐scoured areas frequently lack snow cover and thus may be less vulnerable to earlier snowmelt than areas of deep snow accumulation. Here, we test this expectation by applying black sand to accelerate snowmelt in five 400 m^2^ plots arranged along hillslope gradients. We followed the effects of experimentally accelerated snowmelt on snow‐off timing, growing season length, soil moisture, and plant community composition for 6 years. The largest changes in snow‐off timing (average of 15 days earlier) and growing season length occurred in areas that accumulated the most snow, but there was no effect on soil moisture during the growing season. While plant community composition varied spatially along ambient gradients in snow depth, experimental early snowmelt caused only limited directional changes in composition through time. There was no evidence of homogenization of plant community composition across snow depth gradients. However, species climate niche traits (range in climatic water deficit and range in snow affinity) and specific leaf area explained some variation in species responses to the treatments over time. Taken together, areas that accumulate the most snow are likely to be the most sensitive to changing snowmelt, driving changes in snow‐off timing and growing season length. While we did not detect taxonomic shifts in the plant community over the course of 6 years, some traits can explain variation in plant responses, showing that changing environmental conditions are shifting plant strategies.

## Graphical abstract

We experimentally accelerated snowmelt along gradients of natural snow accumulation to test if deep snow areas are more sensitive to early snowmelt. Some environmental variables responded to experimentally accelerated snowmelt like snow‐off timing and growing season length; this did not translate into consistent changes to the plant community composition.

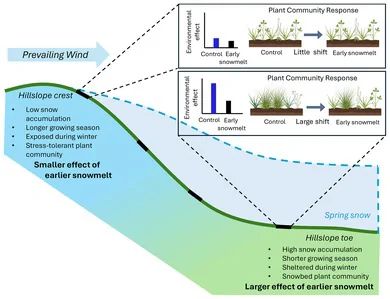

## 1 Introduction

From changing temperatures and precipitation patterns to increasing frequency and intensity of extreme events, understanding how climate change will affect the biodiversity and functioning of ecosystems is a major challenge (Grimm et al. 2013; Parmesan 2006). Mountain ecosystems, which represent one fifth of the Earth's land surface and harbor unique plant communities that contribute substantially to global biodiversity (Körner 2024) and water provisioning (Barnett et al. 2005; Immerzeel et al. 2019), are experiencing especially fast rates of climate change (Grabherr et al. 2010; Oldfather et al. 2025). These systems are characterized by small‐scale variation in topography and microclimate (Jiménez‐Alfaro et al. 2024). Macroclimate changes may be decoupled from the microclimates experienced by plants (Dobrowski 2011), depending on local topography, resulting in a mosaic of patches that are more or less vulnerable to large‐scale climate change (Kulonen et al. 2018; Opedal et al. 2015). Microclimate refugia that buffer portions of the landscape from climate change exposure may be an important mechanism that mediates extinction risk (Maclean et al. 2015; Malanson et al. 2024; Suggitt et al. 2018).

Winter snow accumulation plays a critical role in structuring biodiversity and determining function in cold ecosystems (Chen et al. 2008; Litaor et al. 2008; Niittynen, Heikkinen, and Luoto 2020). Changing temperatures and precipitation regimes also determine snow accumulation dynamics (Fyfe et al. 2017; Huss et al. 2017; Musselman et al. 2021), resulting in a general trend toward earlier snowmelt timing across mountain regions worldwide (Barnett et al. 2005; Elias et al. 2021; Klein et al. 2016; Rixen et al. 2022). Previous research on the effects of earlier snowmelt shows that an earlier initiation of growing season, shifts in plant phenology (Frei and Henry 2022; Jerome et al. 2021; Rose‐Person et al. 2024), damage due to exposure to early cold or wind (Larcher et al. 2010; Neuner 2014; Wheeler et al. 2014), and a longer growing season (Jabis et al. 2020; Livensperger et al. 2016; Semenchuk et al. 2016) are all common effects of earlier snowmelt. These changes are likely to affect plant productivity and composition, alter plant‐insect interactions, and ultimately shift ecosystem functioning in mountain ecosystems (Rixen et al. 2022).

Snow accumulation patterns vary over sub‐meter spatial scales (Sturm 2015) due to the combination of topography and prevailing winds that redistributes snow. This mosaic of deep (meter+) to shallow (centimeters) snow cover and bare patches results in large variation in plant exposure to both cold temperatures overwinter and sunlight in spring. Thus, changes in snow regime could lead to heterogeneous responses across small scales (Suding et al. 2015; Wipf et al. 2009). For example, on wind‐scoured ridge tops with no to little snow (Litaor et al. 2008), changes in precipitation and temperature will have little effect on snow accumulation patterns and the timing of snowmelt (Jay et al. 2023; Kudo et al. 2010; Wieder et al. 2017). Alternatively, areas of higher snow accumulation, such as sheltered zones on the lee side of ridges or vegetation, may see larger effects from changes in precipitation and temperature. In these sheltered areas, where snow depth is typically greatest, earlier snowmelt timing could have a much greater impact on environmental conditions and plant responses (Blankinship et al. 2014; Harpold 2016). However, other changes to snow characteristics with changing climate could result in other patterns like more homogeneous snowpack due to heavier snow that is not redistributed by wind as easily (Badger et al. 2021). Experiments like snow fence studies have been able to examine the effects of additional snow accumulation, often resulting in vegetation change and ecosystem responses (Smith et al. 2018; Wipf and Rixen 2010).

The distribution of snow has strong impacts on plant distributions and abundance. Plant growth in areas with deep snow is often limited by the very short snow‐free growing season. In these areas, earlier snowmelt might lengthen the time plants have available to grow, resulting in increased biomass and favoring more competitive species (Carbognani et al. 2012). However, longer growing seasons might also expose plants to drier, hotter conditions, or damaging wind and freeze events, which can have negative effects on current snowbed species that are adapted to cool, moist conditions (Matteodo et al. 2016). While it is uncertain whether earlier snowmelt will primarily benefit species that are drought‐tolerant or those that can grow most rapidly with an earlier start to the growing season, we expect that the magnitude of both environmental and plant responses to earlier snowmelt will be greatest where more snow naturally accumulates, as those are the places that can experience the greatest possible change in snow conditions. This hypothesis has rarely been tested in the literature (Verrall et al. 2023).

Where responses to earlier snowmelt are spatially variable, plant community composition at the landscape level could become less heterogeneous under earlier snowmelt. In alpine systems, there is often almost complete plant compositional turnover between areas that accumulate the most and the least snow across the landscape (Winkler et al. 2018), generating high plant beta diversity (Opedal et al. 2015). Earlier melt timing may lead to plant community homogenization if environmental conditions become more similar across snow depth gradients and snowbed specialist species are replaced with species adapted to low snow conditions (Matteodo et al. 2016; Niittynen, Heikkinen, and Luoto 2020). Decreasing beta diversity may reduce alpine resilience to future climate change if the relatively more homogeneous plant community results in plant phenotypes adapted to a narrower range of conditions (Graae et al. 2018; Kulonen et al. 2018).

Changes to plant community composition and homogenization might be predicted by plant strategies. Snow conditions are closely related to plant strategies in alpine tundra (Choler 2005; Verrall et al. 2023), where the presence and amount of snow are strong determinants of functional and phylogenetic diversity (Rissanen et al. 2023). Areas with little snow are often dominated by species that are tolerant of extreme cold and dry conditions, such as cushion plants, while species in areas with deep snow tend to be those that can rapidly take advantage of short growing seasons by having evergreen leaves or very fast growth rates (Happonen et al. 2019; Spasojevic and Suding 2012). These strategies can be expressed in terms of plant functional traits such as plant height, leaf size, etc. (Bjorkman et al. 2018; Niittynen, Heikkinen, and Luoto 2020). Leaf economic and plant stature traits predict species responses to experimentally increased snow where taller species with cheaper leaf tissue (higher specific leaf area, SLA lower leaf C) were found to increase in abundance (Henn et al. 2024). Thus, we would expect that earlier snowmelt should favor species with lower SLA and shorter stature, especially in areas with high baseline snow accumulation (Happonen et al. 2019; Niittynen, Heikkinen, and Luoto 2020; Venn et al. 2011; Verrall et al. 2023). Plant bioclimatic niches and niche breadths that are the result of long‐term adaptation to different climate conditions across species ranges may also predict species responses to changing climate conditions (Afkhami et al. 2020; Lynn et al. 2021; Mäkinen et al. 2025). For example, plant climatic niche characteristics predicted long‐term changes in species abundances where species from colder regions increased where there is little snow (Oldfather et al. 2023). Other studies have shown that plant thermal niches either do not respond to, or lag substantially behind, rates of climate change (Mäkinen et al. 2025; Pacheco‐Riaño et al. 2023) with consistent trends toward losing thermal generalists (Mäkinen et al. 2025).

**FIGURE 1:**
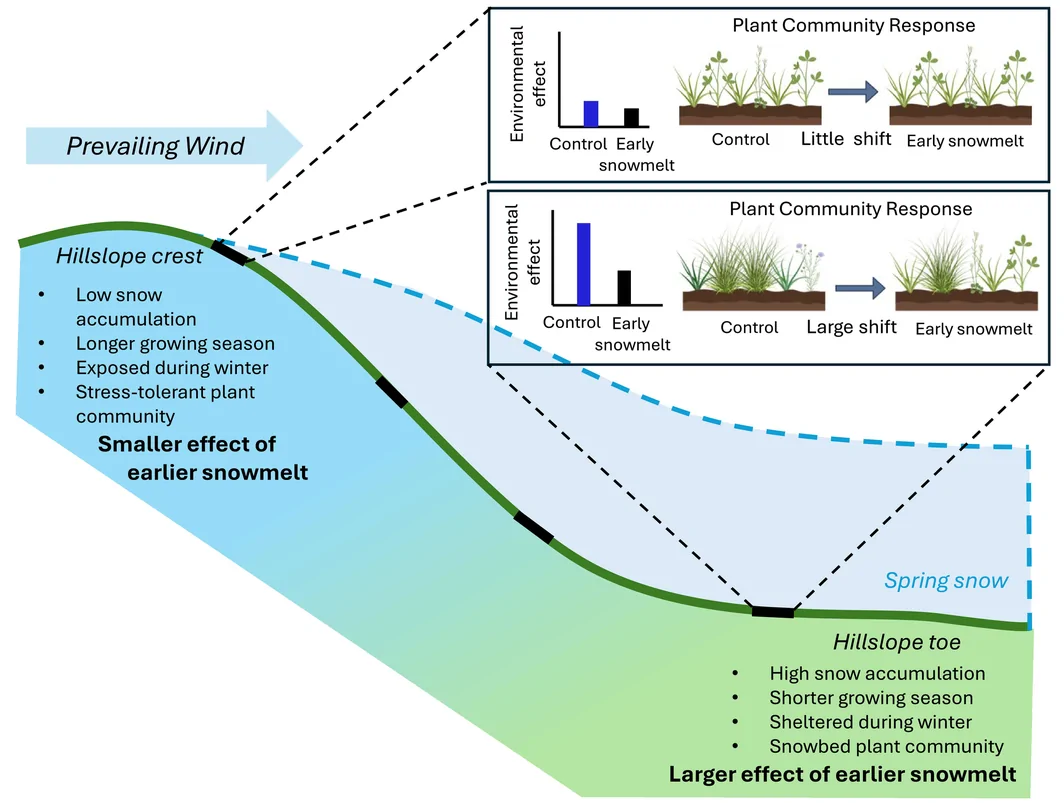
Expected effects of earlier snowmelt along topographical gradients in snow accumulation in alpine settings. The amount of snow accumulated during winter impacts many environmental characteristics in each area. We expect that early snowmelt will have a greater effect on environmental conditions in areas that naturally accumulate more snow (hillslope toes) compared to areas with unreliable snow cover (hillslope crests). We also expect that plant communities in deep snow areas will shift more and begin to resemble upslope communities while hillslope crest areas will see less change in response to earlier snowmelt, resulting in a loss of beta diversity as plant communities along the snow depth gradient become less distinct. Black lines along the hillslope indicate environmental and plant community monitoring locations and inserts at the hillslope crest and toe are representations of our expected results.

A better understanding of how topographic heterogeneity influences alpine plant sensitivity to earlier snowmelt is critical for predicting how plant diversity will be affected as winter conditions continue to change in mountain regions. While there is a great deal of research on plant responses to changing snow patterns (Rixen et al. 2022; Wipf and Rixen 2010), relatively little research has specifically examined environmental and plant responses across existing snow depth gradients within a given site. In this experiment we added black sand to large plots that span natural snow accumulation gradients to increase the rate of snowmelt and measured a suite of environmental and plant responses. Our goal was to better understand the extent to which natural gradients in snow depth influence how earlier snowmelt affects environmental conditions, such as growing season length and moisture availability, and how this ultimately affects plant communities (Figure 1). Specifically, we asked four questions: (1) How does the effect of earlier snowmelt timing on environmental variables (e.g., soil moisture and temperature) depend on snow accumulation patterns? (2) Does plant community composition in areas with deeper snow respond more to earlier snowmelt compared to areas that accumulate less snow? (3) Do plant communities become more homogeneous under earlier snowmelt? and (4) Can the responses of individual species to earlier snowmelt be predicted by either climate niche or morphological traits? Overall, we expect that areas with deep snow will show the greatest change in environmental conditions and that plant community composition will shift in a way that will increase homogenization across the snow depth gradients. We also expect that species with traits related to warm and dry climate niches and stress tolerance will be more successful when snowmelt occurs earlier.

## 2 Methods

### 2.1 Study Sites

We conducted this study in the alpine tundra at Niwot Ridge. Located in the Front Range of the Southern Rocky Mountains in Colorado, USA (40°03′N, 105°35′W), this site is characterized by a cold, high elevation climate (Mean Annual Temperature: −2.1°C, Mean Annual Precipitation: 1000 mm) and average summer temperatures have increased by about 0.08°C per year over the last two decades (Oldfather et al. 2023). Average snow accumulation ranges from 0 to 450 cm in any given year with a slight decreasing trend, especially in areas with the highest snow accumulation (Oldfather et al. 2023). The site has high topographic heterogeneity that relates to snow accumulation and plant community composition (Spasojevic et al. 2013). In sheltered areas where snow accumulates, snowbed plant communities dominate relatively deep soil. On ridgetops where snow rarely accumulates, fellfields are present with very rocky soils and sparse plant cover primarily composed of mainly cushion plants such as *Silene acaulis* . In intermediate conditions, there are moist and dry meadow communities that have higher graminoid cover, dominated by *Kobresia myosuroides* in xeric areas and *Deschampsia caespitosa* in more mesic areas (Litaor et al. 2008). The growing season is short with the last snowfall typically occurring in late May, peak plant growth in July, and the first snowfall in September with an average of 47 frost‐free days per year (Komárková and Webber 1978).

The five sites included in our experiment range in elevation from 3381 to 3514 m. All five sites contain alpine tundra; the two lowest elevation sites have some krummholz trees near or in the sites while the three higher elevation sites contain no woody species (Table S1). Each site was chosen to span a local crest‐to‐toe topographic gradient, where snow accumulation and moisture varied within sites based on wind exposure and drainage patterns (Figure S1).

In 2018, we established two adjacent 10 × 40 m study plots at each of the five study sites. Plots were oriented with their long side parallel to the slope gradient (Figure S1) to span a natural snow accumulation gradient from hillslope crest to hillslope toe (Figure 1). One plot at each site was randomly assigned to an early snowmelt treatment, while the other served as a control (Blankinship et al. 2014). The early snowmelt treatment consisted of manually dispersing a thin layer of black sand consisting of mostly silica dioxide (WAXIE Sanitary Supply, Denver, CO, USA) at a rate of 227 kg sand per 10 × 40 m plot in the spring of each year after the suspected last snowfall event (date range: May 4—June 13, Forrester et al. 2024). This black sand decreases the albedo of the plot to increase solar radiation absorption and speed snowmelt without modifying the total amount of snow in each plot and has been successfully used in previous experiments at our site and elsewhere (Blankinship et al. 2014; Bueno de Mesquita et al. 2020). It thus alters snowmelt rate and timing without altering precipitation or water inputs. Furthermore, our experimental treatments functioned similarly to dust‐on‐snow events, which are a major contributor to snowmelt throughout the Rocky Mountains (Naple et al. 2025). Following snowmelt, we applied the same amount of black sand on the control plot of each site (date range: Jun 30—August 23, Forrester et al. 2024). The large size and crest‐to‐toe placement of the 10 × 40 m plots were selected to minimize lateral snowmelt runoff from outside the treatment area. While small plots are often used in snow removal studies due to their more tractable size (Wipf and Rixen 2010), our large‐scale plots aimed to ensure that the hydrology, soil temperatures, and light conditions accurately represent larger‐scale changes in snow regimes.

To evaluate environmental responses within each treatment plot, we installed continuous soil moisture and temperature sensors at four positions along the slope gradient in the midline of each plot (Meter Group Eros 12 sensors; 5 cm depth, Figure S1; Morse et al. 2024). Similarly, snow depth was measured using snow probes at five locations positioned along the midline. Snow depth measurements were collected approximately weekly following an initial measurement on the date of black sand application for each plot. Based on these snow depth measurements, we calculated the maximum snow depth at each measurement point for each year as a covariate in our models. Erroneous (e.g., spikes, sensor failure) data in the soil temperature and moisture time series were removed prior to analysis (Morse et al. 2024).

To evaluate plant community responses, we recorded plant species composition annually in eight 1 × 0.5 m subplots arranged along the slope gradient with a pair of plant composition subplots located at approximately the same elevation as each environmental data logger (Figure S1). Plant species composition was quantified using point intercept methods, where all species touching a vertically dropped pin flag stem were recorded at 50 evenly spaced points in each subplot. Any species present but not hit at a point were given an abundance of 0.5 (Smith et al. 2023). The total duration of the experiment was 6 years, with plots established in summer 2018, the first of 6 annual black sand applications occurring in spring 2019, the last black sand application occurring in spring 2024, and the final plant and soil measurements collected in summer 2024.

### 2.2 Species Traits

We used species functional traits to evaluate whether species' strategies are useful in explaining which species responded to early snowmelt. This included commonly measured leaf and plant stature functional traits including leaf area, SLA, leaf dry matter content, leaf C:N ratio, leaf carbon 13 isotope ratio (a proxy for water use efficiency), and plant vegetative height. These traits were measured on species across Niwot Ridge between 2008 and 2018 according to standard protocols described in (Pérez‐Harguindeguy et al. 2013; Spasojevic et al. 2022). Prior to analyses, highly skewed traits (leaf area, SLA, and plant vegetative height) were log‐transformed.

To determine how species climate niche might relate to their responses to early snowmelt, we quantified species temperature and climatic water deficit tolerances based on geographical occurrence records from the Global Biodiversity Information Facility (GBIF 2023) and global climate from the TerraClimate dataset (Abatzoglou et al. 2018). After filtering species occurrence records to remove duplicates, records co‐located with biodiversity institutions, and records found at sea using the CoordinateCleaner package in R (Zizka et al. 2019), we used Google Earth Engine (Gorelick et al. 2017) to derive mean annual temperature and climatic water deficit information for each occurrence point in North America. We then calculated species‐specific climate niche values for both the average and range of mean annual temperature and climatic water deficit across the occurrence range of each species (Oldfather et al. 2023).

We also calculated a snow depth affinity metric for species in the study to evaluate how snow niche might relate to responses to early snowmelt based on long term species composition and snow depth measurements from the Saddle at Niwot Ridge (Walker, Morse, et al. 2025; Walker, Humphries, et al. 2025), a well‐researched site that encompasses crest‐to‐toe gradients in snow depth. To calculate this snow affinity metric, we first extracted the maximum snow depth for each year and each plot of the Saddle grid then z‐transformed the snow depth measures within each year to relativize values across years that vary in total snowfall. We then averaged all the values across years for each plot to determine a relativized, average maximum snow depth for each plot. Finally, we calculated the mean and standard deviation in snow depth across all plots where each species was observed to determine whether species are restricted to plots with more or less snow, or whether a species is a generalist across snow depth gradients.

### 2.3 Quantifying Environmental and Plant Community Responses to Early Snowmelt Treatment

To quantify how the early snowmelt treatment influences environmental conditions, we derived the following from the environmental monitoring described above: timing of snow‐off, heat accumulation, and soil dryness. We expect that these metrics should be the most responsive to changing snowmelt timing and should affect plant responses during the growing season. To calculate the timing of snow‐off, we determined the day of year of the first observation of snow depth where there was no snow present at a given snow monitoring point. This corresponds to the end of snowmelt. For heat accumulation, we calculated the soil heat accumulation as accumulated degree days where the mean daily soil temperature was > 2°C from January 1 up until September 7 of each year. We excluded days after September 7, aligning with the timing with which plants generally senesce and become dormant, and because there is high interannual variation in the timing of soil freezing and snowfall at the end of the growing season. We also excluded data from sensors in any year where there were fewer than 250 days of valid measurements to remove sensors that had large amounts of missing data (57 out of 173 locations across sites and years were removed). Finally, we quantified the frequency of dry soil events as the proportion of days during the growing season each year (May 1–September 15) with average soil volumetric water content below 0.13, as this is a threshold known to relate to plant water stress in the type of soil at Niwot Ridge LTER (Billings and Bliss 1959; Reed et al. 2024).

To quantify responsiveness at the plant community level, we calculated Bray‐Curtis dissimilarities and differences in functional diversity over time (Cardoso et al. 2015; Petchey and Gaston 2002) for each subplot relative to that subplot's value during the first year of the experiment. We used the first year of the experiment as a comparison because we did not have pre‐treatment data. We acknowledge this decision could result in underestimation of the treatment effects. We also used the complete Bray–Curtis dissimilarity matrix for all subplots in all years to perform a Principal Coordinates Analysis to visualize the distribution and shifts in plant community composition across sites and through time. We analyzed changes in taxonomic and functional beta diversity at the site level by calculating the average pairwise dissimilarity using the *beta.multi* function in the BAT package (Cardoso et al. 2015) between all vegetation subplots at a given site and treatment in each year and compared these to the average dissimilarity in the first year. To quantify response at the species level, we calculated the log ratio of abundance in the last year compared to the abundance in the first year for each species that was present in at least 20 plot‐year combinations across the experiment. For the community and species analyses, we used only the last year of data compared to the first because (1) we expected that the dissimilarity and changes in abundance should accumulate through time and (2) it is simpler to fit and interpret single‐year models than full time series. This modeling choice might affect our results if the first or last years had unusual weather, but visual assessment of responses across all years does not suggest that this is an issue. We include the community analysis results for all years in the supplemental materials (Figures S2–S5).

### 2.4 Data Analysis

Prior to analysis, we scaled and centered the maximum snow depth variable to help with model fitting and interpretation of parameter estimates. To examine the effects of early snowmelt treatment and maximum snow depth on environmental and plant responses, we used linear mixed effects models. For environmental responses, snow‐off timing, heat accumulation, and soil dryness were the response variables with treatment, maximum snow depth, and their interaction as fixed explanatory variables. For random effects, we included subplot nested in treatment plot, nested in site to account for repeated measures of the same subplots and the nested structure of our measurements, along with year to account for inherent differences in weather and other factors between years.

We also used linear mixed effects models to examine plant community and species responses. For the community responses (community composition and functional diversity change), we included the same model structure as described above except omitting the year variable because we included just the final year in these analyses. Because our beta diversity analyses examine variation of all subplots within a given site and treatment combination, we did not include maximum snow depth as a fixed effect, and we included only site as a random intercept. For our species‐specific responses, our fixed effects included maximum snow depth, treatment, trait value of interest, and all possible interaction terms. Our random effects included the nested plot structure (random intercept) and species identity (random intercept and slope relative to treatment) to account for differences between species there were unrelated to the trait of interest.

All analyses were conducted in R version 4.5.1 (RCoreTeam 2022). We used the *EDIutils* package (Smith 2023) for data access, *tidyverse* (Wickham et al. 2019) for data wrangling and visualization, and the *emmeans* package (Lenth 2023) to calculate posthoc tests of differences in model estimates. For linear mixed effects models, we used the *lmerTest* package in R (Kuznetsova et al. 2017) with denominator degrees of freedom estimated using Satterthwaite's method.

## 3 Results

### 3.1 Environmental Responses to Early Snowmelt Timing

**FIGURE 2:**
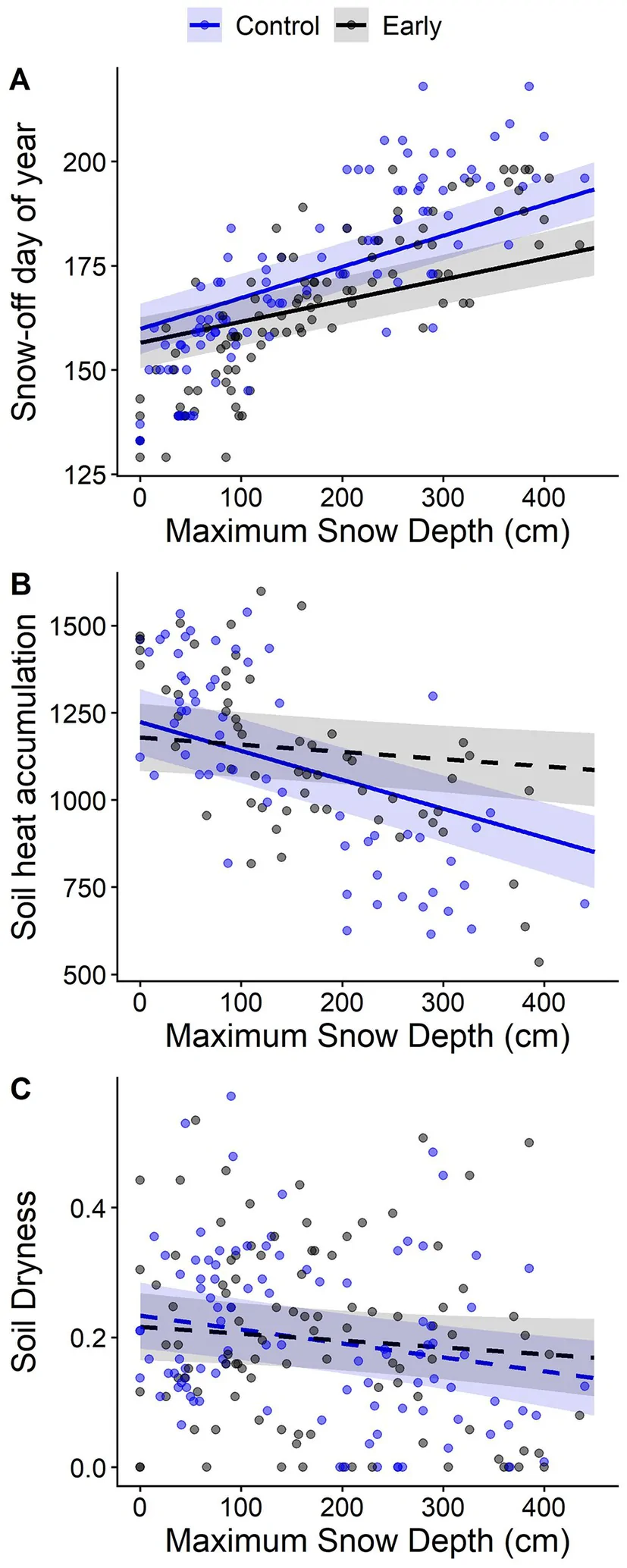
Environmental responses to early snowmelt timing and natural snow depth gradients for snow‐off timing (A), heat accumulation (B, soil temperature degree days where mean soil temperature was greater than 2°C), and soil dryness (C) (the proportion of days each season where average volumetric water content (VWC) was less than 0.13). Colored dots indicate measured values while lines and error bars indicate the trends from generalized linear mixed models. Dashed lines indicate non‐significant effects of snow depth on the response while solid lines indicate significant effects.

Abiotic response variables responded to treatment and in some cases, the response differed by maximum snow depth (Figure 2, Table 1). As expected, the date of snow‐off was later in areas with deeper snow in both the control and treatment plots, and early snowmelt treatment plots were free of snow earlier than control plots on average (Table 1, treatment effect). While the strength of the relationship between the snow depth and snow‐off timing was only marginally different between the treatment and control plots (ANOVA, *F* = 2.84, df = 1, 56.6, *p* = 0.1), the smaller magnitude of the snow‐depth and snow‐off timing relationship seen in the treatment plots indicates that the impact of the treatment on snowmelt timing was greatest in areas with more snow. Heat accumulation, measured as temperature degree days before September 7, declined as maximum snow depth increased. However, the effect of snow depth was weaker in the treatment plots, indicating that the early melt treatment increased heat accumulation most in areas with the deepest snow (Figure 2, Table 1). Finally, the proportion of days with mean soil volumetric water content less than 0.13 showed no strong responses to snow depth or our treatment. Control plots showed a marginally significant response to snow depth (*t*‐test, *t*‐ratio = −1.7, df = 108, *p* = 0.09) indicating that there is a tendency toward wetter conditions in areas with deeper snow, especially in control plots, but there was substantial variation around this trend. The impact of snow‐depth on all abiotic response variables examined was consistently less extreme (slope closer to 0) in the early melt treatment as compared to the control plots, indicating a homogenization of environmental conditions across the snow depth gradient when black sand was applied (Figure 2, Table 1).

**TABLE 1:** Model results for environmental effects of early melt treatment and maximum snow depth. Each cell contains the estimate (*t*‐ratio, *df*) and *p*‐value. Treatment effect refers to the difference in intercept estimates, which is the equivalent of the difference between treatments at the average snow depth. Snow depth effect refers to the slope of the response along the snow depth gradient and the “difference in snow depth effect” refers to whether the slope estimates differ between control and treatment plots. In all cases, the *p*‐values indicate tests of whether the estimate differs from zero and bolded cells indicate a *p* < 0.05 and italicized cells indicate a *p* < 0.1. Estimates are based on models with centered and scaled variables so are not on the same scale as the values graphed in Figure 2.

Response	Treatment effect	Snow depth effect (Control)	Snow depth effect (Early)	Difference in snow depth effect
Date of snow‐off	**−0.37 (−3.4, 12) 0.005**	**0.43 (6.7, 89) < 0.0001**	**0.29 (4.1, 78) 0.0001**	*0.14 (1.7, 57) 0.1*
Heat accumulation	0.2 (1.5, 3) 0.23	**−0.35 (−5.3, 77) < 0.0001**	−0.09 (−1.2, 72) 0.24	**−0.26 (−3.1, 42) 0.004**
Soil dryness	0.001 (0.06, 33) 0.95	*−0.03 (−1.7, 116) 0.09*	−0.01 (−0.8, 108) 0.4	−0.01 (−0.7, 86) 0.5

### 3.2 Plant Community Composition, Functional Diversity, and Beta Diversity

**FIGURE 3:**
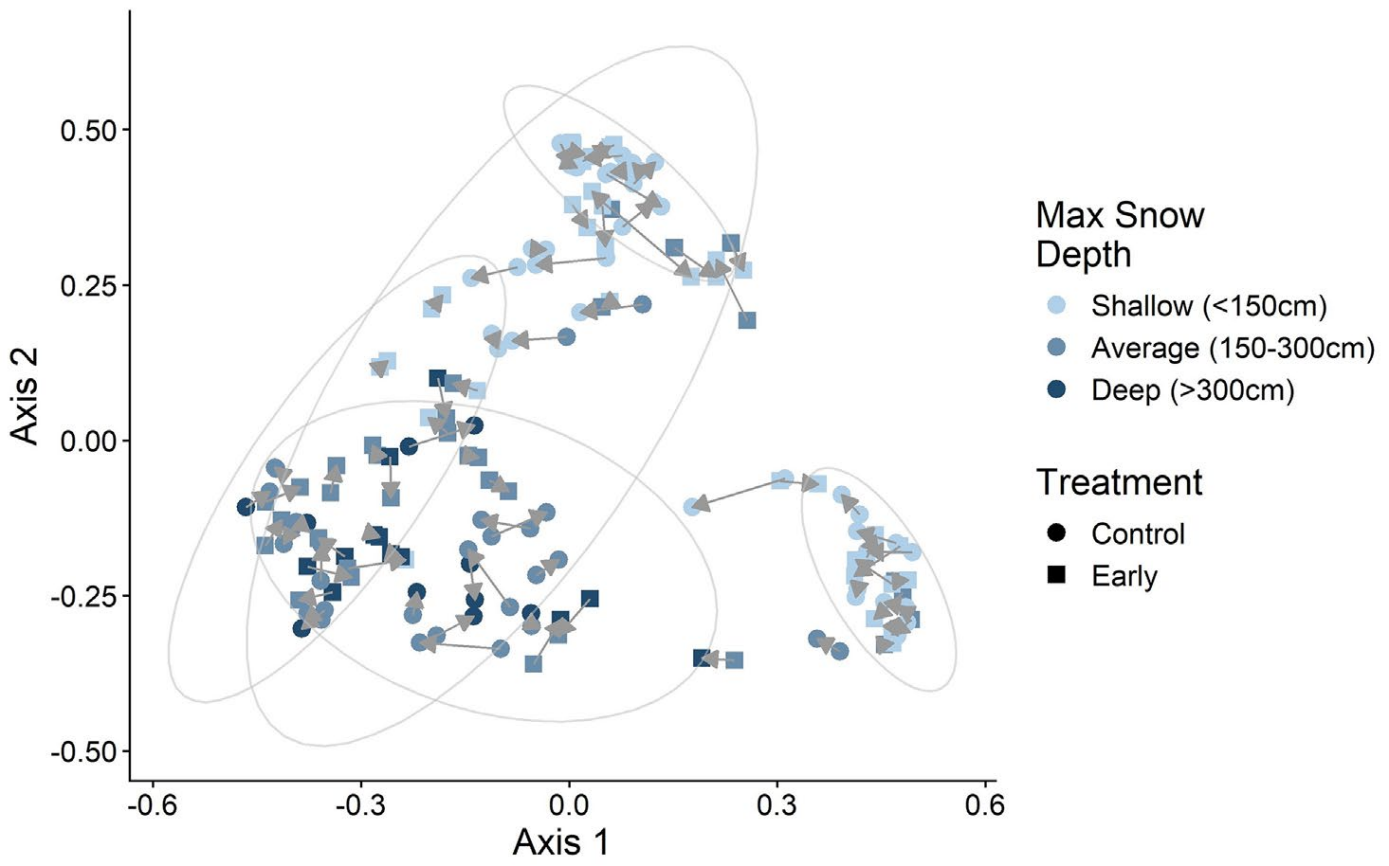
Principal coordinates analysis of plant community composition across the subplots measured in this project. Color of the points indicate the maximum snow depth category. Point shape indicates treatment type and points at the base of an arrow are from the first year of the experiment while points at the end of the arrows are the same plots at the end of the experiment. Gray ellipses indicate the 95% confidence interval for the plots at each study site to illustrate how plant species composition varies within and between the five study sites.

Plant community composition varied among sites and across snow depths, but not between treatments (Figure 3). Shifts in plant community composition from the first to last year were highly variable, without consistent direction or distance.

**FIGURE 4:**
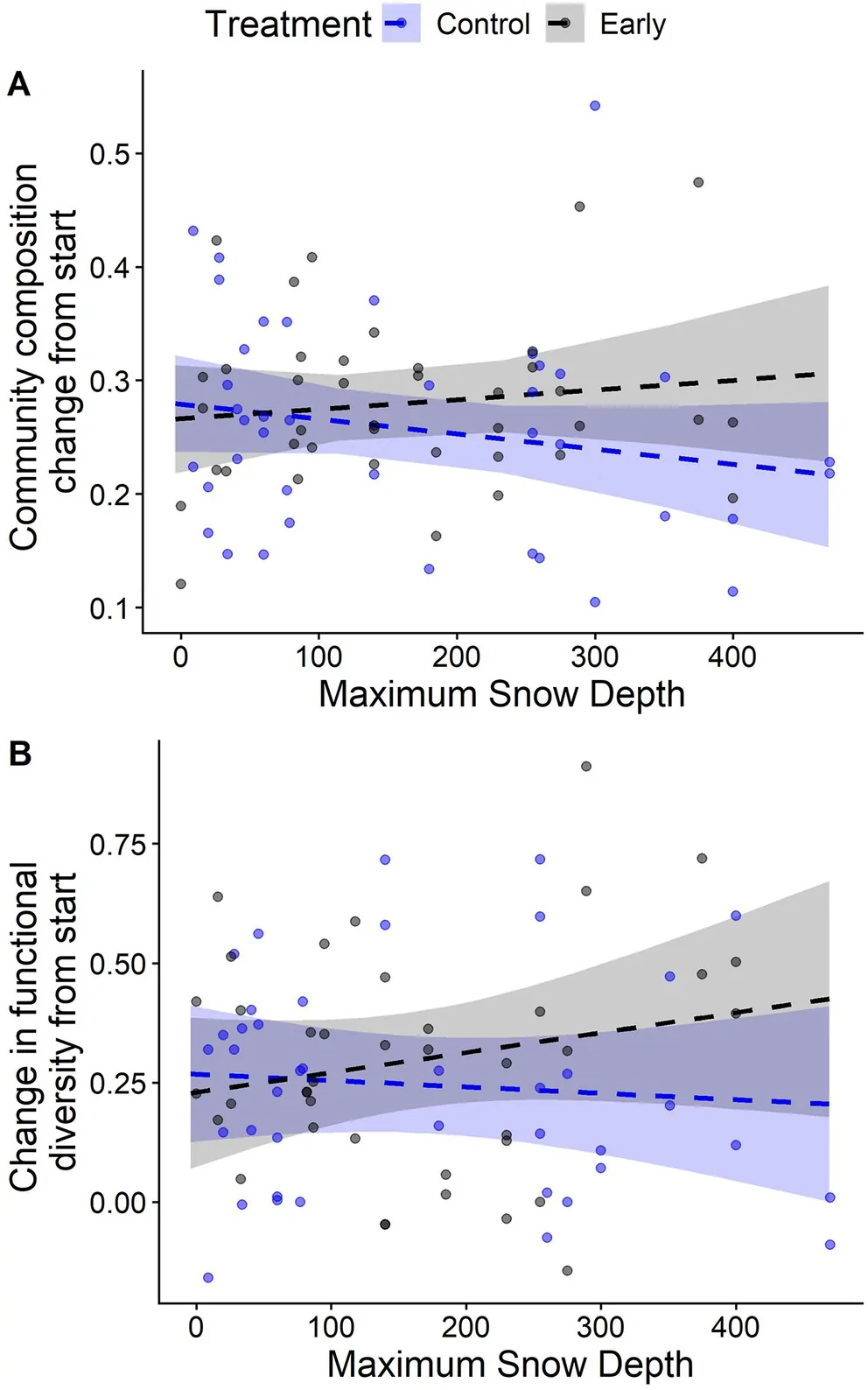
Multivariate difference in (A) species taxonomic community composition (Bray–Curtis dissimilarity) and (B) functional diversity between the first year and the last year along the maximum snow depth gradient. Point, line, and error bar colors correspond to the treatments. Dashed lines indicate non‐significant effects of snow depth on the response while solid lines indicate significant effects. For each year of the experiment plotted separately, see Figures S2 and S3.

Community composition change (Bray–Curtis dissimilarity) from the start to the end of the experiment did not vary with snow depth (Control slope = −0.016 ± 0.01, *p* = 0.18; treatment slope = 0.01 ± 0.01, *p* = 0.48; difference in slope *p* = 0.16, *t*‐ratio = −1.4, df = 76; Figure 4A). Similarly, there were no significant differences in functional diversity responses to maximum snow depth or treatment (Control slope = −0.016 ± 0.04, *p* = 0.65; treatment slope = 0.05 ± 0.04, *p* = 0.26; difference in slope *p* = 0.17, *t*‐ratio = −1.4, df = 74; Figure 4B).

**FIGURE 5:**
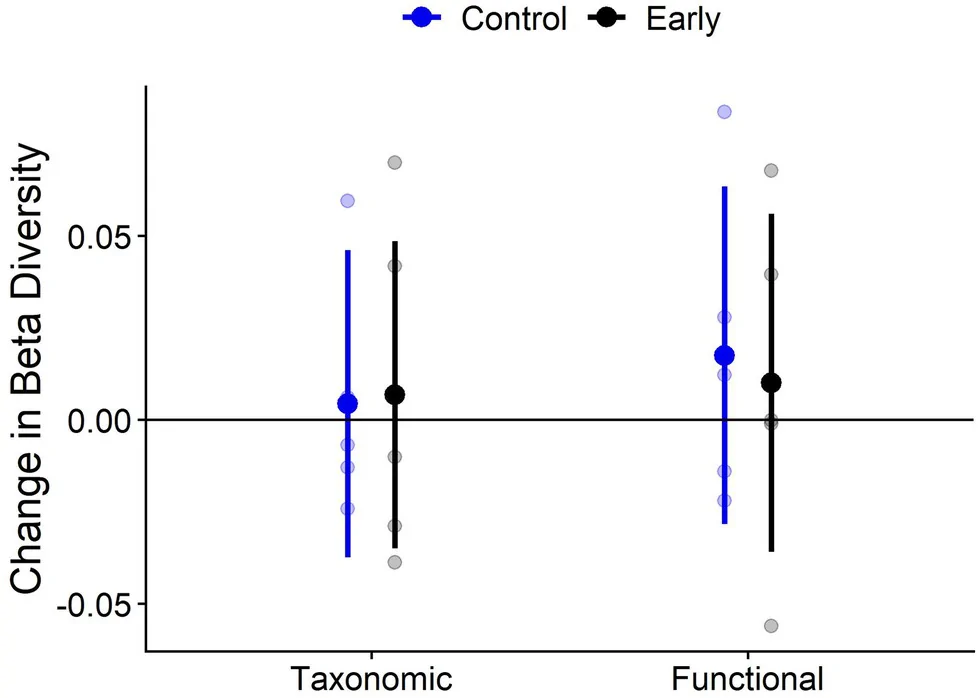
Difference in beta diversity (average of all pairwise Bray–Curtis dissimilarities within a site‐treatment‐year combination) for the final year of the experiment compared to the first year. Small points indicate different sites colored by treatment. Large points and error bars indicate the mean and standard error around that mean for each treatment. The *y*‐axis values are a proportional change relative to the beta diversity value of a given site treatment combination in the first year where negative values indicate homogenization while positive values indicate heterogenization through time. For all years of the experiment separated, see Figures S4 and S5.

Taxonomic and functional beta diversity within treatments at a given site did not consistently change with any model predictors (*p* > 0.1 for all tests; Figure 5). Average change was very close to zero for both treatment and control plots and both kinds of beta diversity. Some sites showed decreasing beta diversity, indicating homogenization, but just as many sites showed increasing beta diversity, indicating increasing variation in taxonomic and functional composition.

### 3.3 Trait‐Mediated Species Responses

**FIGURE 6:**
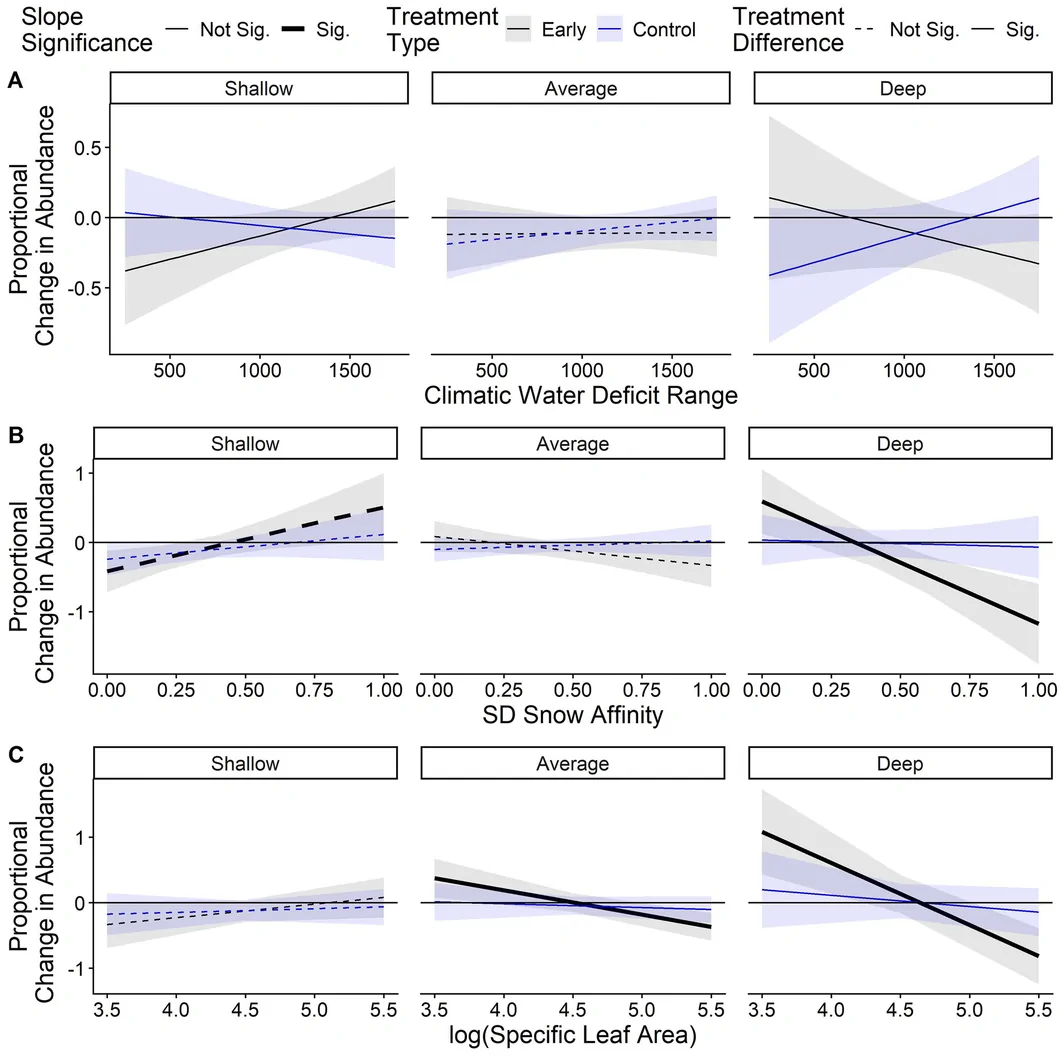
Trait‐mediated responses. Panels correspond to traits that showed significant interactions in overall models (Table 2) where panel A is range in climatic water deficit, panel B is the standard deviation of snow affinity, and panel C is specific leaf area. Each panel shows the effect of a trait on change in abundance across species in different snow depth classes (shallow = 0 cm maximum snow depth, average = 233 cm maximum snow depth, deep = 450 cm maximum snow depth, based on 95% percentiles and average of maximum snow depth measures). Line width indicates whether slope estimates differ from zero, where narrow lines indicate that slope estimates do not differ from zero (Not Sig., *p* > 0.05) and thicker lines indicate that the slope estimate differs from zero (Sig., *p* < 0.05). Line colors indicate plot treatment type. Line type indicates whether the slopes of each treatment differ significantly from control slopes where solid lines indicate that treatment slopes differ (Sig., *p* < 0.05) and dashed lines indicate that treatment slopes do not differ from control slopes (Not Sig., *p* > 0.05).

Two of the six bioclimatic niche characteristics we examined (range in snow affinity and climatic water deficit) were predictive of species responses to snow treatment (Table 2, Figure 6). Surprisingly, early melt treatments tended to reduce the abundance of climatic water deficit specialist species (species with a narrow range in climatic water deficit niches) in areas with little snow compared to control treatment plots, while the opposite was marginally true at deep snow levels (Figure 6A). On the other hand, the early melt treatment resulted in increasing abundances of snow specialists at the expense of snow generalists in the deep snow areas (Figure 6B). Of the six morphological traits we tested, only SLA was predictive of species responses to the snow treatment. Species with low SLA increased in abundance in middle and deep snow depths in early melt treatment plots (Figure 6C).

**TABLE 2:** ANOVA results for models testing species‐trait effects on abundance change. Climate niche traits are reported first, followed by leaf/plant structure traits. Values in each cell correspond to an F‐statistic (numerator DF, denominator DF), *p*‐value. Tests with *p*‐values less than 0.05 are bolded and those with *p*‐values less than 0.1 are italicized. Plant traits that had significant interactions between snow, treatment, and the trait values are plotted in Figure 6.

Term	Climatic water deficit	Climatic water deficit range	Temperature	Temperature range	Snow depth	Snow depth standard deviation
Treatment	0.34 (1, 47) 0.56	0.13 (1, 34) 0.72	1.32 (1, 37) 0.26	0.25 (1, 31) 0.62	0.13 (1, 19) 0.72	0.64 (1, 145) 0.42
Max Snow Depth	0 (1, 657) 0.96	0.03 (1, 426) 0.87	0.35 (1, 28) 0.56	0 (1, 235) 0.98	**8.72 (1, 59) < 0.001**	**8.95 (1, 94) < 0.001**
Trait	0.18 (1, 54) 0.67	0.65 (1, 39) 0.43	0.89 (1, 49) 0.35	0.99 (1, 39) 0.33	1.55 (1, 21) 0.23	0.11 (1, 138) 0.74
Treatment × Max Snow Depth	0.22 (1, 519) 0.64	**4.05 (1, 359) 0.04**	0.02 (1, 553) 0.9	0.12 (1, 459) 0.72	0.12 (1, 247) 0.73	*3.31 (1, 832) 0.07*
Treatment × Trait	0.14 (1, 55) 0.71	0.05 (1, 33) 0.83	1.77 (1, 52) 0.19	0.12 (1, 29) 0.74	0.34 (1, 20) 0.57	0.83 (1, 139) 0.36
Trait × Max Snow Depth	0 (1, 723) 0.95	0.09 (1, 524) 0.77	0.22 (1, 502) 0.64	0.06 (1, 678) 0.81	**10.62 (1, 421) < 0.001**	**12.28 (1, 801) < 0.001**
Treatment × Max Snow Depth × Trait	0.48 (1, 530) 0.49	**5.56 (1, 326) 0.02**	0.49 (1, 409) 0.48	0.55 (1, 428) 0.46	0.73 (1, 344) 0.39	**6.32 (1, 885) 0.01**

Term	Specific leaf area	Leaf dry matter content	D13C	C:N	Plant vegetative height	Leaf area
Treatment	*2.76 (1, 73) 0.1*	0 (1, 28) 0.97	2.41 (1, 60) 0.13	0.46 (1, 20) 0.51	0.02 (1, 32) 0.88	0.36 (1, 37) 0.55
Max snow depth	**9.58 (1, 449) < 0.001**	0.4 (1, 384) 0.53	0.36 (1, 370) 0.55	0 (1, 402) 1	0.21 (1, 269) 0.64	0 (1, 21) 0.97
Trait	*2.83 (1, 54) 0.1*	0.4 (1, 30) 0.53	1.84 (1, 26) 0.19	2.28 (1, 24) 0.14	0.04 (1, 37) 0.85	0.37 (1, 37) 0.54
Treatment × Max Snow Depth	**4.77 (1, 477) 0.03**	0.98 (1, 243) 0.32	0.01 (1, 601) 0.92	0.18 (1, 267) 0.67	0.78 (1, 377) 0.38	0.4 (1, 655) 0.53
Treatment × Trait	*2.8 (1, 68) 0.1*	0 (1, 28) 0.96	2.35 (1, 59) 0.13	0.31 (1, 21) 0.58	0.14 (1, 30) 0.72	0.25 (1, 30) 0.62
Trait × Max Snow Depth	**9.56 (1, 508) < 0.001**	0.54 (1, 397) 0.46	0.37 (1, 384) 0.54	0.01 (1, 421) 0.93	0.31 (1, 627) 0.58	0.09 (1, 730) 0.76
Treatment × Max Snow Depth × Trait	**5.02 (1, 480) 0.03**	0.75 (1, 226) 0.39	0.01 (1, 602) 0.91	0.19 (1, 242) 0.67	0.55 (1, 393) 0.46	0.52 (1, 831) 0.47

## 4 Discussion

Trends in snow cover loss have been linked to dramatic effects in alpine ecosystems, leading to longer growing seasons and shifting the phenology, composition, and productivity of alpine vegetation in some cases (Rumpf et al. 2022). Results from our snowmelt manipulation emphasize that on a local scale, the impacts of declining snow cover are distributed unevenly across the landscape. Overall, the greatest effect of experimentally accelerated snowmelt for two environmental responses (date of snow‐off and heat accumulation) occurred in areas that naturally accumulate more snow, likely because these are areas where snow‐off timing has the greatest potential to advance. These environmental changes resulted in homogenization of the growing season environment across the snow depth gradients, but plant community composition did not show consistent diversity or homogenization responses. We did, however, see evidence that trait‐mediated species responses to early snowmelt were generally strongest in deep snow areas, possibly because species living in high snow accumulation areas may be most sensitive to a loss of snow.

The black sand treatment had the strongest effect on snow‐off date in areas with the deepest snow, where it advanced the snow‐off date by ~15 days on average. This difference is similar to the difference in snow‐off timing generated in other experiments in similar systems that used black fabric or black sand to increase the rate of snow melt (Blankinship et al. 2018; Bueno de Mesquita et al. 2020; Leonard et al. 2020; Steltzer et al. 2009) and to the variation in average snow‐off date between years (about 20 days from earliest to latest average snow‐off date) during the study period. However, this advancement is approximately double the typical treatment effect from snow manipulation treatments that remove snow by shoveling (Rixen et al. 2022). In parallel with the black sand effects on snow‐off date, the black sand treatment affected heat accumulation most at average to deep snow depths where the early melt treatment had more accumulated GDDs compared to control plots, likely due to earlier snow‐off dates. Conversely, in areas with low snow accumulation, there was no treatment effect on snow‐off date or heat accumulation. Our soil dryness response did not show strong responses to maximum snow depth or treatment type. We expected that earlier snow‐off would result in drier soils (Blankinship et al. 2018), and we did see a slight trend toward drier soils in areas with less snow. However, the negative relationship between snow depth and soil moisture was observed in both treatment and control plots, indicating that advancing snowmelt timing by applying black sand did not change soil moisture. The lack of expected pattern in soil dryness could be due to a range of factors including hydrological variation and connectivity within plots that we did not capture in our monitoring, supplemental precipitation during the growing season, and the soils in high snow accumulation areas may be able to retain enough moisture that earlier melt has little effect later in the season.

There is ample evidence that snow depth plays a key role in determining species composition in tundra ecosystems and that variation in snow depth results in species composition turnover over small scales (Niittynen, Heikkinen, Aalto, et al. 2020; Rissanen et al. 2023; Winkler et al. 2018). We also observed high turnover in species composition spatially across the snow depth gradients at our study sites. While we would have also expected evidence of changing species composition over time, particularly in the areas that saw the largest changes in environmental conditions (the deeper snow in the early melt treatment), only a modest non‐significant trend was evident. Other experiments have found that snow manipulation often results in changes in species composition (Wahren et al. 2005), although these are largely snow addition experiments and studies that examine plant phenological responses (Wipf and Rixen 2010). Given the long lifespans of most tundra plants, it is not surprising that it may take more than 6 years to see strong community shifts in response to changing snow conditions.

Beta diversity across the entire hillslope gradient of our experimental plots also did not change consistently with early snowmelt, suggesting that plant community changes in deep snow areas may not shift towards the species assemblages of low snow plant communities. Other research at Niwot Ridge has shown that environmental changes do not necessarily favor species that would be expected to benefit from these changes (Bueno de Mesquita et al. 2024), suggesting that plant responses to a range of global change drivers may result in novel communities (García Criado et al. 2025). Previous research has also shown that changes in plant communities over time can be non‐directional even given directional environmental change (Spasojevic et al. 2013). Turnover resulting in homogenization along the snow depth gradient is likely slow because it relies on both mortality and recruitment, both of which are slow processes in tundra plant communities. Recruitment is especially rare in established communities where competition is strong (Margreiter et al. 2021) and the effects of changing climate conditions on alpine plant seed germination and establishment are understudied (Briceño et al. 2015).

Plant strategies ranging from leaf traits to bioclimatic niches explain variation in plant species responses to global changes at this study site (Henn et al. 2024; Huxley et al. 2023; Oldfather et al. 2023) and in arctic and alpine tundra vegetation in general (Bjorkman et al. 2018; Jónsdóttir et al. 2023; Soudzilovskaia et al. 2013). We also found that functional traits explained species responses to early snow‐off across the snow depth gradient. Of those traits that did significantly predict species responses, niche ranges were more important than average niche descriptors. For example, we found that taxa with broad climatic water deficit ranges tended to decrease in deep snow areas in the early melt treatment while average climatic water deficit niche was not important (Table 2). The importance of narrow niche ranges suggests that species specialized on specific water conditions are more successful under early melt in deep snow areas. Variation in snow affinity was also important while average snow affinity was not. Snow affinity specialists were strongly favored in deep snow under early melt conditions, suggesting that deep snow specialists in those plots were already present and are able to take advantage of longer growing seasons to increase their growth when snow melts earlier, although previous research has shown that snowbed species do not necessarily respond in predictable ways to earlier or later snowmelt timing (Bueno de Mesquita et al. 2024; Galen and Stanton 1995). While snowbed species can persist or even thrive for some time under earlier snowmelt scenarios in the short term due to less energy limitation, over longer terms non‐snowbed species may move in and outcompete snowbed specialists (Hülber et al. 2011). These results are also notable because narrow ranges in climatic tolerances often translate to narrower geographical ranges (Cardillo et al. 2019; Yu et al. 2017) and potentially higher sensitivity to climate change (Thuiller et al. 2005).

While species niche characteristics were more predictive of treatment effects than plant leaf and stature traits, one trait, SLA, stood out with significant relationships to abundance change under earlier snowmelt. In deep‐snow areas, species with low SLA increased in abundance when snow melted earlier, which we also saw in monitoring plots at the same research site (Oldfather et al. 2023). Low SLA is characteristic of more resource‐conservative species on the leaf economics spectrum (Díaz et al. 2016; Wright et al. 2004), which might indicate that earlier snowmelt generates more stressful environmental conditions for plants in deep‐snow habitats.

Taken together, our results show that the responsiveness of environmental and plant changes in response to snow‐off timing varies across naturally occurring topographic gradients in snow depth. Specifically, plant communities in areas that accumulate substantial amounts of snow each winter are showing shifts in some functional traits in response to earlier snow‐off, but this responsiveness diminishes in areas that accumulate less snow. Interestingly, the observed changes in high‐snow plant communities did not suggest that earlier snow‐off will lead them to converge with low‐snow communities despite changes to environmental responses that reflect homogenization across snow accumulation gradients. Our data highlight how a trait‐based approach can be useful for understanding plant species' responses to environmental change over time and may provide a first hint at plant community composition responses. The lack of homogenization of plant communities across snow depth gradients may be a sign of the resilience of tundra plant communities to changing snow accumulation and melt patterns and contribute to the unexpected pattern of relative stability in satellite‐based metrics of tundra vegetation change across much of the tundra despite rapid climate change (Callaghan et al. 2021). We need more long‐term studies to better understand whether change is slow and to better understand the resistance of tundra plant communities to climate change.

## Author Contributions

**Jonathan J. Henn:** conceptualization, formal analysis, writing – original draft. **Emily C. Farrer:** conceptualization, methodology, writing – review and editing. **Katherine Bardsley:** data curation, formal analysis, writing – review and editing. **Nancy C. Emery:** formal analysis, investigation, funding acquisition, project administration, resources, supervision, writing – review and editing. **Katharine N. Suding:** conceptualization, methodology, project administration, resources, funding acquisition, writing – review and editing, supervision. **Chiara Forrester:** conceptualization, investigation, methodology, project administration. **Jennifer F. Morse:** data curation, investigation, writing – review and editing, project administration. **Sarah C. Elmendorf:** conceptualization, data curation, investigation, methodology, writing – review and editing. **Jane G. Smith:** conceptualization, data curation, investigation, methodology, project administration, writing – review and editing. **Clifton P. Bueno de Mesquita:** conceptualization, investigation, methodology, writing – review and editing. **Marko J. Spasojevic:** conceptualization, investigation, methodology, writing – review and editing.

## Funding

This work was supported by the National Science Foundation (DEB‐1637686, DEB‐2224439, DGE‐2022138).

## Conflicts of Interest

The authors declare no conflicts of interest.

## Supporting information


**Figure S1:** Map of study site locations at Niwot Ridge Long Term Ecological Research site in Colorado, USA and the layout of sampling locations within study plots.
**Figure S2:** Bray–Curtis dissimilarity from first year (Year 0, 2018) for all subsequent years of the study.
**Figure S3:** Difference in functional diversity from first year (Year 0, 2018) for all subsequent years of the study.
**Figure S4:** Difference in beta diversity of all subplots in a given site compared to the first year for all following years.
**Figure S5:** Difference in functional beta diversity of all subplots in a given site compared to the first year for all following years.
**Table S1:** Characteristics of each site. TPI = topographic position index calculated for a 30‐m diameter surrounding vegetation monitoring plots.

## Data Availability

All data used in these analyses are publicly available in Environmental Data Initiative repositories (environmental data loggers: Morse et al. (2024), Snow depth: Forrester et al. (2024), Plant species composition: Smith et al. (2023), Plant traits: Spasojevic et al. (2022)) and code used to analyze the data are available here: https://doi.org/10.5281/zenodo.21903524.
