# Supplementary material for: Natural Snow Accumulation Gradients Mediate Environmental and Plant Trait Responses to Earlier Snowmelt in Alpine Tundra
Source: Glob Chang Biol. 2026 Aug 24;32(8):e71068. doi: 10.1111/gcb.71068 (PMC13502219; doi:10.1111/gcb.71068)
Supplement: Supplementary file 1 [file GCB-32-e71068-s001.docx]

**Supplemental Materials**

Table 1: Characteristics of each site. TPI = topographic position index calculated for a 30-meter diameter surrounding vegetation monitoring plots.

| Site | Elevation (MASL) | Aspect (Direction) | Slope  (%) | Above Treeline? | Minimum TPI | Maximum TPI |
| --- | --- | --- | --- | --- | --- | --- |
| Audubon | 3471 | ENE | 23 | Yes | -0.65 | 0.44 |
| East Knoll | 3514 | SE | 25 | Yes | -0.32 | 0.26 |
| Lefty | 3451 | NE | 30 | Yes | -0.62 | 0.61 |
| Soddie | 3381 | SSE | 43 | No | -0.28 | 0.53 |
| Trough | 3413 | SSE | 22 | No | -1.14 | -0.24 |

Figure S1: Map of study site locations at Niwot Ridge Long Term Ecological Research site in Colorado, USA and the layout of sampling locations within study plots.

Figure S2: Bray-Curtis dissimilarity from first year (year 0, 2018) for all subsequent years of the study.

Figure S3: Difference in functional diversity from first year (year 0, 2018) for all subsequent years of the study.

Figure S4: Difference in beta diversity of all subplots in a given site compared to the first year for all following years.

Figure S5: Difference in functional beta diversity of all subplots in a given site compared to the first year for all following years.
